# Capacity Fade Analysis of Sulfur Cathodes in Lithium–Sulfur Batteries

**DOI:** 10.1002/advs.201600101

**Published:** 2016-07-21

**Authors:** Jianhua Yan, Xingbo Liu, Bingyun Li

**Affiliations:** ^1^ BiomaterialsBioengineering and Nanotechnology LaboratoryDepartment of OrthopaedicsWest Virginia UniversityMorgantownWV26506USA; ^2^ Department of Mechanical and Aerospace EngineeringWest Virginia UniversityMorgantownWV26506USA

**Keywords:** electrolytes, fundamentals, Li–S batteries, poly‐shuttle

## Abstract

Rechargeable lithium–sulfur (Li–S) batteries are receiving ever‐increasing attention due to their high theoretical energy density and inexpensive raw sulfur materials. However, their rapid capacity fade has been one of the key barriers for their further improvement. It is well accepted that the major degradation mechanisms of S‐cathodes include low electrical conductivity of S and sulfides, precipitation of nonconductive Li_2_S_2_ and Li_2_S, and poly‐shuttle effects. To determine these degradation factors, a comprehensive study of sulfur cathodes with different amounts of electrolytes is presented here. A survey of the fundamentals of Li–S chemistry with respect to capacity fade is first conducted; then, the parameters obtained through electrochemical performance and characterization are used to determine the key causes of capacity fade in Li–S batteries. It is confirmed that the formation and accumulation of nonconductive Li_2_S_2_/Li_2_S films on sulfur cathode surfaces are the major parameters contributing to the rapid capacity fade of Li–S batteries.

## 1 Introduction

Rechargeable batteries are used in applications ranging from portable electronic devices to automobiles. Li‐ion batteries have become prominent due to their high energy density compared with other battery technologies.^1^ However, there is a continued demand for improvements in the cost, energy, power, and safety of these Li‐ion batteries.^2^ Li–S batteries have shown higher energy density and lower cost compared to Li‐ion batteries.^3^ Li–S batteries are of particular interest for stationary and electrical vehicle applications where high capacities and size reduction are important. However, the efficiency and cycle life of the Li–S batteries need to be improved to enable their use in practical applications.^4^

A prominent issue is associated with the fairly complex chemistry occurring on the cathode.^5^ The ultimate discharge products are insoluble and nonconductive Li_2_S_2_/Li_2_S; however, there are a number of intermediate soluble products with different Li to S ratios (Li_2_S*_x_*, 3 ≤ *x* ≤ 8).^6^ For one thing, the nonconductive sulfur (S_8_) and Li_2_S_2_/Li_2_S greatly decrease the utilization of the active sulfur materials and pose major issues for power capability.^7^ For another, the accumulation of insoluble Li_2_S_2_/Li_2_S on cathode surface decreases the electrochemical reaction sites in the cathode thus resulting in capacity fade.^8^ Another issue is related to the intermediate soluble polysulfide species, which can diffuse from the cathode to the electrolyte, and thus reduces the overall quantity of sulfur in the cathode, leading to a decreased battery capacity.^9^ They can further diffuse to the Li‐anode, where they are reduced to nonconductive and insoluble Li_2_S_2_/Li_2_S and deposit on the Li‐anode surface. Part of these deposits may react with the following arrived soluble species and generate more soluble species, which can diffuse back to the cathode.^10^ The uncontrollable deposition layers consume active sulfur materials and increase battery resistance, resulting in rapid capacity fade of batteries, while the shuttle circulates between the two electrodes leading to self‐discharge, making the charging time of the battery toward infinity, greatly decreasing the efficiency and cycle life of batteries.

Various strategies have been developed to enhance the stability of Li–S batteries in recent years.^11^ However, systematic studies on capacity fades of Li–S batteries are rarely reported. Li–S battery is a liquid electrochemical system, in which the amounts of electrolytes play an essential role in battery performance. On one hand, dissolution of soluble polysulfide species makes it easy for electron‐transfer and Li‐ion diffusion in cathode, and thus promotes a complete reaction and a high charge/discharge rate.^12^ On the other hand, the dissolution causes the loss of sulfur materials into the electrolytes, and leads to capacity fade. Therefore, the ratio of sulfur‐to‐electrolyte should be properly balanced. In this study, we examined the capacity fade of binder‐free cathodes in Li–S batteries by adopting different amounts of electrolytes. The cathodes were made of sulfur multi‐walled carbon nanotube (SMCNT) composites with carbon nanofiber (CNF) current collectors. A detailed discussion on capacity fade was provided. By examining the electrochemical performance, sulfur reaction kinetics, and electrochemical impedance spectroscopy (EIS), the major reason for the rapid capacity fade was concluded as the formation of thick layers of insoluble and nonconductive Li_2_S_2_/Li_2_S observed films.

## 2 Theoretical Capacity Fade Analysis of Li–S Batteries

It is known that sulfur undergoes a multistep reaction during each discharge and charge process. To better understand the roles of each factor on the capacity degradation, where the capacity from and the effects of multistep reactions between the various polysulfide species should be clearly understood.

**Figure 1:**
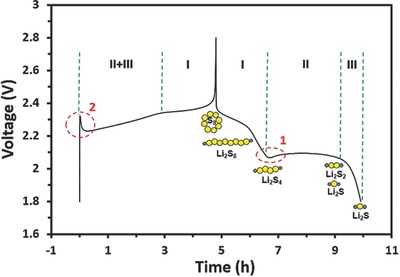
A typical initial voltage profile of Li–S battery employing SMCNT cathode at a discharge and charge current rate of 0.2 C.

A typical voltage profile of Li–S battery is plotted in **Figure** **1**. In discharge, sulfur is reduced to Li_2_S by accepting the Li‐ions and electrons at cathode; in charge, a reversed reaction takes place, as shown in Equation (1)[[qv: 9a]]

(1) $$S_{8}+16\;{\mathrm{Li}}^{+}+16\;e\Leftrightarrow 8\;{\mathrm{Li}}_{2}S$$

It is well accepted that the discharge process can be divided into three regions:

Region I: Initially, the solid‐state sulfur is dissolved into the liquid electrolyte and forms liquid‐state sulfur, which is further reduced to S^2−^_4_ , as shown in Equation (2)

(2) $$S_{8}\left(s\right)\Rightarrow S_{8}\left(l\right)+4\;e\to 2S_{4}^{2-}$$

This was a half‐electron charge transfer per sulfur atom, contributing about 25% of the total sulfur capacity.^13^ The controlled Nernst equation is shown in Equation (3)[[qv: 9a]]

(3) $$E_{U}=E_{U}^{\theta }+\frac{RT}{n_{H}F}\ln\frac{\left[S_{8\left(l\right)}^{0}\right]}{{\left[S_{4}^{2-}\right]}^{2}}$$

With increasing depth of discharge (DOD), the concentration of soluble S^2−^_4_ species increases, while S_8_ is almost maintained at its saturated concentration because of its low solubility in liquid electrolytes. Consequently, the voltage in this region decreases all the time, and it is mainly affected by the concentration of S^2−^_4_. Once the electrolyte viscosity rises to a certain level, the Li‐ion transport encounters difficulties, as verified by the small reverse peak that is circled as point 1 (Figure 1). Thus, the rapid voltage drop in region I reflects concentration polarization.

Region II: Next, the soluble species S_4_^2−^ are further reduced to insoluble Li_2_S_2_ or Li_2_S, as shown in Equations (4.1) and (4.2)[[qv: 9b]]

(4.1) $$S_{4}^{2-}+8\;{\mathrm{Li}}^{+}+6\;e\to 4\;{\mathrm{Li}}_{2}S$$

(4.2) $$S_{4}^{2-}+4\;{\mathrm{Li}}^{+}+2\;e\to 2\;{\mathrm{Li}}_{2}S_{2}$$

Some literature suggests that the transition from the high to the low discharge voltage plateau happens concurrently with the start of the formation of solid Li_2_S_2_ and Li_2_S.^6^, ^14^ This stage contributes to the major portion of the capacity with a fixed voltage. The controlled Nernst equation is shown in Equation (5)[[qv: 9a]]

(5) $$E_{L}=E_{L}^{\theta }+\frac{RT}{n_{L}F}\ln\frac{\left[S_{4}^{2-}\right]}{{\left[S_{2}^{2-}{\mathrm{/S}}^{2-}\right]}^{4}\;}$$

Since both Li_2_S and Li_2_S_2_ have extremely low solubility in liquid electrolyte, the concentrations of S^2−^_2_ and S^2−^ are constant.^15^ On the other hand, S^2−^_4_ decreases gradually in concentration due to the slow kinetic reactions from soluble S^2−^_4_ to insoluble and nonconductive Li_2_S_2_/Li_2_S. Therefore, the discharge curve stays between 2.1 and 2 V for a long time. Until the cathode is largely covered by the nonconductive Li_2_S_2_/Li_2_S, which greatly increases the cell resistance and blocks charge transfer paths, the voltage shows a quick drop, and the reaction is terminated.

Region III: The last sloping tail corresponds to a solid‐to‐solid reduction from Li_2_S_2_ to Li_2_S, as shown in Equation (6)

(6) $${\mathrm{Li}}_{2}S_{2}+2\;{\mathrm{Li}}^{+}+2\;e\to 2{\mathrm{Li}}_{2}S$$

The conversion of Li_2_S_2_ to Li_2_S is the most difficult due to the slowness of solid‐state diffusion in the bulk.^16^

In the charge process, a long, flat, low plateau is seen first, representing the oxidation of insoluble Li_2_S_2_/Li_2_S to soluble long‐chain polysulfide species. A reduced polarization during the charge process caused by the dissolution of solid Li_2_S_2_/Li_2_S is verified by the small peak that is circled as point 2 (Figure 1).^4^ The upper charge plateau indicates the oxidation reactions from the soluble long‐chain polysulfide species to solid sulfur.

During the charge/discharge cycling, soluble polysulfide species move freely through the separator to the Li‐anode and multiple concurrently parasitic reactions take place simultaneously.[[qv: 9d,17]] For example, the soluble polysulfide species can react with Li‐ions in the electrolyte and generate insoluble Li_2_S, as shown in Equation (7)

(7) $${\mathrm{Li}}_{2}S_{n}+2\;e+2\;{\mathrm{Li}}^{+}\to {\mathrm{Li}}_{2}S+{\mathrm{Li}}_{2}S_{n-1}$$

This reaction consumes electrolytes and causes capacity fade.

The soluble species may also react with the Li‐metal and form insoluble Li_2_S_2_ and Li_2_S, as shown in Equations (8) and (9)

(8) $$2\;\mathrm{Li}+{\mathrm{Li}}_{2}S_{n}\to {\mathrm{Li}}_{2}S+{\mathrm{Li}}_{2}S_{n-1}$$

(9) $$2\;\mathrm{Li}+{\mathrm{Li}}_{2}S_{n}\to {\mathrm{Li}}_{2}S_{2}+{\mathrm{Li}}_{2}S_{n-2}$$

Both Li_2_S_2_ and Li_2_S easily precipitate onto the Li‐anode surfaces. Moreover, these nonconductive Li_2_S_2_/Li_2_S can continue to react with the soluble species and form more short‐chain soluble species, as shown in Equations (10) and (11), 18

(10) $${\mathrm{Li}}_{2}S+{\mathrm{Li}}_{2}S_{n}\to {\mathrm{Li}}_{2}S_{m}+{\mathrm{Li}}_{2}S_{n-m+1}$$

(11) $${\mathrm{Li}}_{2}S_{2}+{\mathrm{Li}}_{2}S_{n}\to {\mathrm{Li}}_{2}S_{m}+{\mathrm{Li}}_{2}S_{n-m+2}$$

When these short‐chain species become concentrated at the Li‐anode side, they diffuse back to the cathode and are re‐oxidized into long‐chain soluble species. This polysulfide shuttle effect causes self‐discharge and produces current, which does not contribute to charging of the battery and results in a low Coulombic efficiency.

The chemical reactions in Equations (7)–(11) always exist in the liquid‐type Li–S systems, and they are particularly severe in the charge process. In the charge process, the insoluble Li_2_S_2_/Li_2_S can easily transform into soluble long‐chain polysulfide species, but the conversion from the long‐chain polysulfide species to sulfur is very difficult, and some scholars report that polysulfide species do not transform back to elemental sulfur even at 100% depth of charge. With increasing the depth of charging, the concentration of the long‐chain polysulfide species at the cathode becomes larger than that in the anode, thus the diffusion dynamics of these polysulfide species from the cathode to the Li‐anode increases. Meanwhile, the reduction reactions of these migrated polysulfide species on the Li‐anode surfaces are accelerated. Therefore, the second voltage plateau in the charge curve can be seen as a competition between electrochemical oxidation and reduction of the long‐chain polysulfide species.[[qv: 9a]]

The discharge capacity at each DOD is summarized in **Table** **1**. To simplify the analysis, S^2−^_4_ is used to represent the intermediate soluble polysulfide species and Li_2_S_2_/Li_2_S is used as the insoluble discharge products.

**Table 1:** Discharge capacity versus DOD in Li–S batteries

Discharge products	Electrons transferred [mol mol^−1^ S^−1^]	DOD	Specific capacity [mA h g^−1^]
S_8_↔S_4_^2−^	0.5	25%	418
S_8_↔Li_2_S_2_	1	50%	836
S_8_↔Li_2_S	2	100%	1672

The initial discharge capacity (*Q*_i_, mA h g^−1^) can be calculated in Equation (12)

(12) $$\begin{array}{c} Q_{i}=\sum \omega_{n}Q_{s_{8}\to {\mathrm{Li}}_{2}S_{n}}\\ =\omega_{4}\;Q_{s_{8}\to S_{4}^{2-}}+\omega_{2}Q_{s_{8}\to {\mathrm{Li}}_{2}S_{2}}+\omega_{1}Q_{s_{8}\to {\mathrm{Li}}_{2}S\;}\\ =418\omega_{4}+836\omega_{2}+1672\omega_{1} \end{array}$$

where *ω_n_* is the weight percent of S_8_ being converted to S^2−^_*n*_

(13) $$\sum \omega_{n}=1,\;n=1,\;2,\;4$$

Since the reaction from solid Li_2_S_2_ to solid Li_2_S is difficult, *ω*_1_ is far below 1, which is a major factor for the initial discharge capacity loss. On the other hand, the thermodynamic reactions from soluble S^2−^_4_ to insoluble S_8_ during charge is difficult. Therefore, after the initial cycle, most sulfur in the cathode is a soluble polysulfide species in high valence states. From the second cycle, the discharge capacity (*Q_x_*, mA h g^−1^) can be calculated by Equation (14)

(14) $$Q_{x(x>1)}=\sum \omega_{m}^{s_{8}}Q_{s_{8}\to {\mathrm{Li}}_{2}S_{n}}+\sum \omega_{m}^{S_{4}^{2-}}Q_{S_{4}^{2-}\to {\mathrm{Li}}_{2}S_{1,2}}$$

The discharge capacity comprises two parts: one from S_8_ to Li_2_S*_n_* (*n* = 1, 2, 4), and the other from Li_2_S_4_ to Li_2_S_2_/Li_2_S. Since there are still insoluble Li_2_S_2_/Li_2_S at the end of charge, the total weight percent of these two kinds of sulfur is described in Equation (15)

(15) $$\sum \omega_{m}^{s_{8}}+\sum \omega_{m}^{S_{4}^{2-}}=1-\frac{\sum \Delta S_{\left({\mathrm{Li}}_{2}{\mathrm{S+Li}}_{2}S_{2}\right)}}{S}$$

(16) $$\mathrm{where}\;\sum \omega_{m}^{s_{8}}=\omega_{4}^{s_{8}}\;Q_{s_{8}\to S_{4}^{2-}}+\;\omega_{2}^{s_{8}}Q_{s_{8}\to {\mathrm{Li}}_{2}S_{2}}+\omega_{1}^{s_{8}}Q_{s_{8}\to {\mathrm{Li}}_{2}S}$$

(17) $$\sum \omega_{m}^{S_{4}^{2-}}=\omega_{2}^{S_{4}^{2-}}Q_{S_{4}^{2-}\to {\mathrm{Li}}_{2}S_{2}}+\;\omega_{1}^{S_{4}^{2-}}Q_{S_{4}^{2-}\to {\mathrm{Li}}_{2}S}$$

Based on the above analysis, the total capacity fade, *Q*, of the liquid type Li–S battery can be divided into three parts

(18) $$Q=Q_{1}+Q_{2}+Q_{3}$$

where *Q*_1_ is the capacity fade due to the loss of sulfur into the liquid electrolyte; *Q*_2_ is the capacity fade due to the precipitation of nonconductive Li_2_S_2_/Li_2_S films onto the surfaces of both electrodes that form passivation layers, which inhibits further lithiation/delithiation; *Q*_3_ is the capacity fade due to the incomplete conversions from Li_2_S_2_ to Li_2_S in discharge, and from long‐chain polysulfide species to elemental sulfur in charge.

Since Q_1_, Q_2_, and Q_3_ are related to the amounts of electrolytes, to determine their individual influence, three different amounts of electrolytes were adopted in the batteries: 5, 8, and 12 μL mg^−1^, based on sulfur, indicating insufficient, proper, and sufficient quantity of electrolytes, respectively.^19^ Some parameters should be considered when designing a sulfur cathode, in which the conductivity, porosity, and thickness are the most important factors.^20^ In order to reliably track the influence of electrolytes, all other factors should be fixed. In this study, we designed a binder‐free SMCNT cathode with a 3D CNF current collector. The detailed characterization results of the binder‐free SMCNT cathodes are shown in the Supporting Information. Our former studies demonstrated that binder‐free cathodes could reach much higher capacities compared to PVDF‐based cathodes, which limits the access to active sulfur materials and reduces the capacity of Li–S batteries because of the blockage of pores in sulfur cathode caused by PVDF.^21^

## 3 Results and Discussion

### 3.1 Long‐Cycle Performance

**Figure 2:**
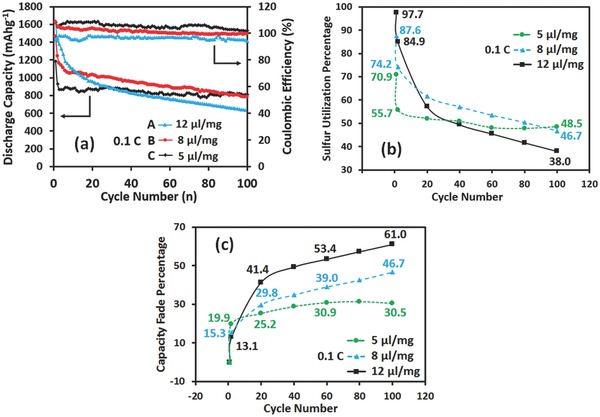
Long‐cycle performance, sulfur utilization, and capacity fade of Li–S batteries employing different amounts of electrolytes. a) Long‐cycle performance at 0.1 C. b) Sulfur utilization versus cycle numbers. c) Capacity fade versus cycle numbers.

The long‐cycle performances and the corresponding capacity fades of the batteries with various amounts of electrolytes are shown in **Figure** **2**. It was observed (Figure 2a) that the batteries with 5 μL mg^−1^ electrolytes had a low capacity and were stable throughout cycling. By contrast, the batteries with 12 μL mg^−1^ electrolytes resulted in a high initial capacity. However, the discharge capacity dropped quickly in the first 30 cycles, and then decreased linearly with cycling. The sulfur utilization and the capacity fade as a function of cycle number are shown in Figure 2b,c, respectively. The sulfur utilization was based on the theoretical sulfur capacity of 1672 mA h g^−1^, while the capacity fade was based on the initial discharge capacity. The capacity was checked on the second cycle, and every 20 cycles after that. As can be seen from Figure 2b, increasing amount of electrolytes in the batteries led to increasing initial discharge capacity, and thus enhanced initial sulfur utilization. At the 100th fully discharged cycle, sulfur utilization for the batteries with 5 and 8 μL mg^−1^ electrolytes showed no obvious difference (48.5% and 46.7%), while the batteries with 12 μL mg^−1^ electrolytes had a sulfur utilization of 38.0%. From Figure 2c, a large irreversible capacity loss between the first two cycles was observed for all of these three batteries, which agreed with the previous analysis. After the second cycle, the capacity fade diverged greatly for these three batteries. The capacity of the batteries with 12 μL mg^−1^ electrolytes decreased rapidly with increasing cycle numbers, and the capacity fade reached 61% over 100 cycles. However, the batteries with 5 μL mg^−1^ electrolytes only lost 30.5% of initial capacity after 100 cycles, and the capacity retention was almost the same with the cycle numbers after 80 cycles. Further, batteries with 12 μL mg^−1^ electrolytes had the fastest rate of capacity fade between the second and the 100th cycle. These results indicated that the amounts of electrolytes had significant influence on capacity and capacity fade of sulfur cathodes.

The batteries with 8 μL mg^−1^ electrolytes always had a Coulombic efficiency (CE) approaching 100% and showed the best combination of capacity and capacity retention. The batteries with 5 μL mg^−1^ electrolytes always had a CE larger than 100%, resulting in a high cycling stability. Of note, the high CE (>100%) means that the discharge capacity was larger than charge capacity, indicating more Li‐ion diffused into the cathode than diffused out of the cathode during cycling. In Li–S battery, Li source is infinite compared to that of sulfur. Insufficient amount of electrolytes resulted in a high concentration of polysulfide species in the cathode structure, and the high viscosity decreased the diffusion of Li‐ion and polysulfide species from cathode to electrolyte, thereby reducing polysulfide shuttle and resulting a high capacity retention. On the other hand, the high viscosity hampered the electronic contact between polysulfide species with conductive CNTs and generated a low discharge capacity. In this study, LiNO_3_ was added into the electrolyte, and thus the batteries with 12 μL mg^−1^ electrolytes also had a high CE of 95%, indicating that the poly‐shuttle effect was limited. However, a low capacity retention after 40 cycles and a rapid capacity fade along the whole cycling process was observed. Therefore, in addition to poly‐shuttle, there should be other reasons for the rapid capacity fade in Li–S batteries.

### 3.2 Sulfur Reaction Kinetics

**Figure 3:**
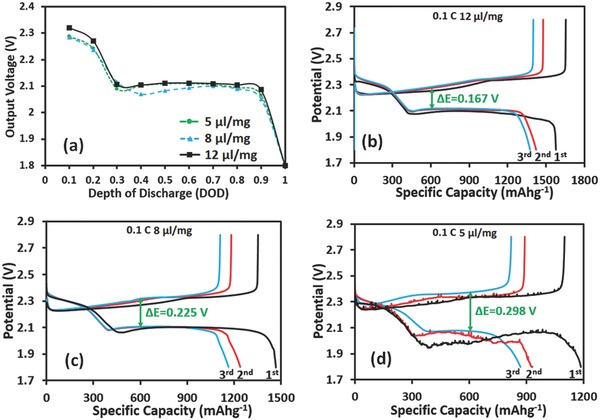
Output voltage and voltage profiles of Li–S batteries with different amounts of electrolytes. a) Output voltages of the batteries over 100 cycles as a function of DOD. b–d) The first three cycles' voltage profiles of Li–S batteries with three different amounts of electrolytes.

**Figure** **3**a shows the output voltage as a function of depth of discharge (DOD); all the batteries had been cycled up to 100 times. The output voltage of the battery under constant current can be simply represented as

(19) $$E=E_{0}-iR_{i}$$

where *E*_0_ is the standard cell potential and *R*_i_ is the internal resistance of the cell, including electrolyte resistance, contact resistance, activation polarizations (*η*_ct_, charge transfer over voltage), and concentration polarizations (*η*_c_) at cathode.^22^ As can be seen, the output voltages of the batteries with 12 μL mg^−1^ electrolytes at each DOD were always larger than those of the other two batteries, indicating that sufficient electrolyte decreased battery resistances and enhanced the reaction kinetics. To further verify this conclusion, the first three cycles' voltage profiles of the Li–S batteries were analyzed, as shown in Figure 3b–d. All of the three batteries showed an increase of both upper and lower discharge plateaus after their initial cycles. The lower voltage plateaus in the initial cycle indicated high polarization of the sulfur cathode during the reaction time. After the initial cycle, most of the active sulfur materials dissolved into the liquid electrolyte and rearranged in the cathode, thus showing an increase in the discharge plateaus. In addition, it was found that an increase in the quantity of electrolyte greatly enhanced the capacities and decreased the voltage difference between charge and discharge plateaus (from 0.298 to 0.167 V), indicating fast reaction kinetics in the batteries with sufficient electrolytes. In the batteries with insufficient electrolytes, a severe polarization (Δ*E* = 0.298 V) and multiple voltage bumps were observed. Insufficient electrolytes slowed or inhibited the transportation of polysulfide species and Li‐ions to CNT surfaces, which caused uneven reactions in the cathode along with the reaction time since the reaction of sulfur can only take place on CNT surfaces, and thus resulted in a rough voltage profiles and high polarization.

As discussed previously, sufficient electrolytes dissolved more soluble polysulfide species and exposed the inner nonconductive sulfur to the conductive CNT framework, driving the reaction forward and thus producing a high capacity. On the other hand, sufficient electrolytes made it easy for polysulfide species to transfer from the cathode to the electrolyte, resulting in sulfur material loss into the dead corner of electrolyte, and thus leading to a rapid capacity fade in the first several cycles. However, the electrolyte most likely would be saturated with the dissolved polysulfide species after several cycles, but the battery capacities always decreased with the 12 or 8 μL mg^−1^ electrolytes (Figure 2a).^15^ Therefore, in addition to the reasons we discussed above, other important reasons may also be responsible for the rapid capacity fades.

### 3.3 Li_2_S_2_/Li_2_S Precipitation Analysis

**Figure 4:**
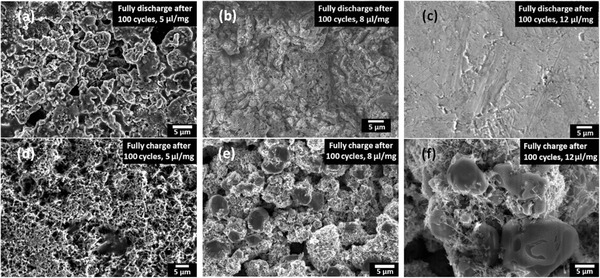
SEM images of the cathodes in Li–S batteries cycled up to 100 times. The SEM images were checked after fully discharged and charged states of batteries employing different amounts of electrolytes. Fully discharged batteries with electrolytes of a) 5, b) 8, and c) 12 μL mg^−1^; and fully charged batteries with electrolytes of d) 5, e) 8, and f) 12 μL mg^−1^.

To further investigate the parameters influencing the capacity fade of Li–S batteries, scanning electron microscope (SEM) was carried out to check the microstructures of the sulfur cathodes cycled up to 100 times (**Figure** **4**). Distinguishable differences between these batteries with different amounts of electrolytes were observed. After being fully discharged to 100 cycles, the cathode samples in the batteries with 5 μL mg^−1^ electrolytes (Figure 4a) were composed of numerous large Li_2_S_2_/Li_2_S particles, among which there were a lot of holes. However, a thick Li_2_S_2_/Li_2_S film with a lot of cracks was observed for the cathode samples of 8 μL mg^−1^ electrolytes (Figure 4b). For the cathode samples of 12 μL mg^−1^ electrolytes (Figure 4c), a thicker Li_2_S_2_/Li_2_S film was observed. The deposition and accumulation of nonconductive and insoluble Li_2_S_2_/Li_2_S films in cathodes led to the structural deterioration of cathodes. On the other hand, after being fully charged to 100 cycles (Figure 4d–f), sulfur agglomerates were seen on the surface of the cathode samples with 8 and 12 μL mg^−1^ electrolytes, while no obvious sulfur accumulations were observed on the samples with 5 μL mg^−1^ electrolytes. In addition, with an increasing amount of electrolytes, the sulfur agglomerate sizes increased. From Figure 4e, it can be seen that the average sulfur agglomerate size was about 5 μm, which is too large for electron transporting. Thus only the sulfur on CNT surfaces could participate in chemical reactions, which resulted in a low output capacity. Based on the SEM results, the formation of nonconductive Li_2_S_2_/Li_2_S films and the formation of large sulfur agglomerates on sulfur cathodes were important factors leading to the rapid capacity fade in Li–S batteries.

### 3.4 Electrochemical Impedance Spectroscopy (EIS) Study

**Figure 5:**
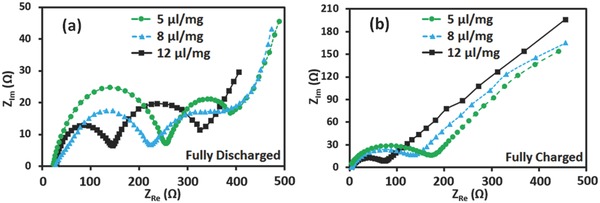
EIS plots of batteries cycled up to 100 cycles with different amounts of electrolytes at a discharge current rate of 0.1 C. The tested batteries had 8 μL mg^−1^ electrolytes. a) Fully discharged states (to 1.8 V). b) Fully charged states (to 2.8 V).

EIS is one of the most powerful tools for studying the electrochemical reactions in batteries.^23^ To elucidate the mechanism of the deposition and dissolution of solid Li_2_S_2_/Li_2_S films, EIS measurements of Li–S batteries cycled up to 100 times were carried out, as shown in **Figure** **5**. In the fully discharged states (Figure 5a), all of the three impedance spectra exhibited two depressed semicircles in high and medium‐frequency regions followed by an inclined line indicating solid‐state diffusion at low‐frequency regions. For comparison, in the fully charged states (Figure 5b), all of the three impedance spectra were composed of one depressed semicircle in high and a short inclined line in low‐frequency regions. It was believed that the semicircle in the high‐frequency region reflected the charge‐transfer process at carbon interface and the semicircle in the medium‐frequency region was related to the formation of solid Li_2_S_2_/Li_2_S films on the CNT surfaces in cathodes.[[qv: 23b]] As can be seen, in the fully discharged state, increasing the quantity of electrolytes increased the resistance of solid Li_2_S_2_/Li_2_S films. In both fully discharged and charged states, the charge‐transfer impedance of the battery with a smaller amount of electrolytes was larger than that of the battery with a higher amount of electrolytes. This result was in agreement with the previous conclusions: sufficient electrolytes led to enhanced charge transfer, but at the same time, produced thicker nonconductive films that increased the resistance of mass transport during the cycles.

**Figure 6:**
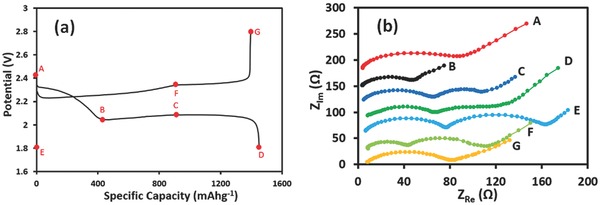
EIS characterization of batteries at the first cycle. The tested batteries had 12 μL mg^−1^ electrolytes. a) The selected points for EIS tests in the voltage profiles. b) EIS spectra at various points.

To examine the formation kinetics of the solid nonconductive Li_2_S_2_/Li_2_S films and large sulfur agglomerates during cycling, EIS studies at various points, as marked with points A–G in **Figure** **6**a, were conducted. The points A–G were selected according to discharge and charge time, in which the discharge/charge time in the first cycle had been used as reference for the calculation of the state‐of‐charge or DOD. The EIS spectra were shown in Figure 6b. Of note, there was a fluctuation with the electrode resistances (characterized by the resistance at the very beginning points in each curve) for different states. As discharge proceeded, sulfur transformed to soluble polysulfide species, and thus the viscosity of electrolyte increased in the cathode, resulting in an increase in cathode resistance. It can be seen from the figures that the impedance spectra could be divided into two types according to the shape of the curves. At points C–F, the EIS spectra comprised two depressed semi‐circles and a straight sloping line; while the EIS spectra at points A, B, and G only presented one depressed semi‐circle and a straight sloping line. These results agreed with the above assumption that the depressed semi‐circle in the medium frequency corresponded to the formation of Li_2_S_2_/Li_2_S films. From A to B, the reduction reaction was dominated by charge transfer resistance, which decreased substantially due to the dissolution of polysulfide species into liquid electrolytes. From B to D, the charge transfer resistance increased gradually due to the slow reaction kinetics from long‐chain polysulfide species to nonconductive Li_2_S_2_/Li_2_S. At the same time, the resistance from the solid Li_2_S_2_/Li_2_S film increased greatly due to the increasing thickness of Li_2_S_2_/Li_2_S films. This result indicated that from B to D, the formation of Li_2_S_2_/Li_2_S films should be a main step controlling the reduction reaction. From E to F, both resistances decreased due to the disappearance of solid Li_2_S_2_/Li_2_S films and the formation of soluble long‐chain polysulfide species. At G, only one depressed semi‐circle that corresponded to the charge‐transfer resistance was observed, indicating that the solid Li_2_S_2_/Li_2_S films were transformed into long‐chain polysulfide species.

**Figure 7:**
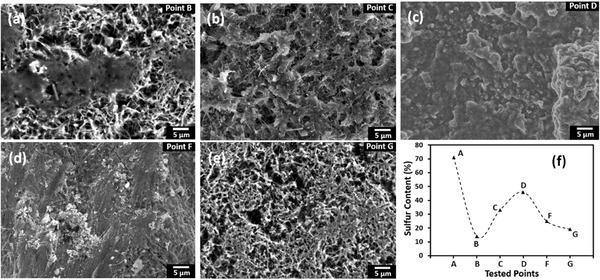
SEM characterization and sulfur content of SMCNT cathodes at various discharge and charge states in the first cycle. The tested batteries had 8 μL mg^−1^ electrolytes. a–e) At discharge points B, C, and D, and charge points E and F. f) Sulfur content at each tested states.

**Figure 8:**
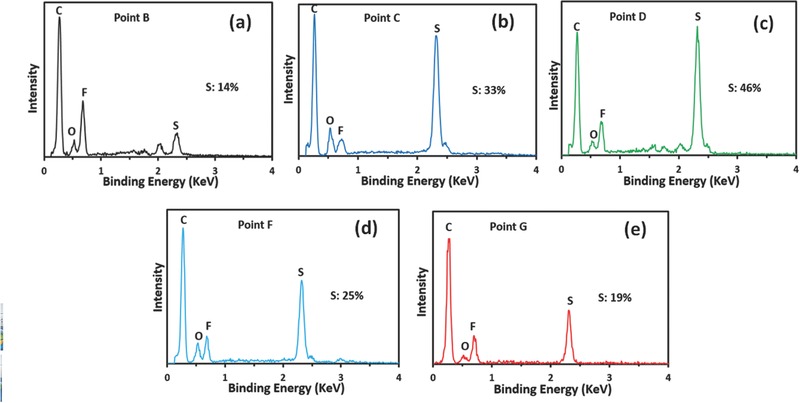
EDX characterization of sulfur cathodes at points a) B, b) C, c) D, d) F, and e) G.

The morphologies and compositions of sulfur cathodes at different discharge and charge states were investigated using SEM and energy‐dispersive X‐ray spectroscopy (EDX), respectively. Before conducting SEM tests, the cycled cathodes at different states were washed with dioxolane (DOL) solution in the glove box. During this process, the soluble long‐chain polysulfide species were completely removed, and only solid insoluble species remained on the cathode. Then the washed cathodes were dried in the glove box for 24 h. Prior to discharge, the original SMCNT cathodes had well‐distributed sulfur particles (Figure S1c, Supporting Information), and the initial sulfur content was 71%. After discharge to point B, as can be seen from **Figure** **7**a, most sulfur particles disappeared, and only minimal solid sulfur materials were observed. When it comes to the middle point C of the lower discharge plateau, there were disconnected solid films on the surfaces of CNTs without any aggregated sulfur particles (Figure 7b). Until the end of the discharge, the CNT surfaces were fully covered with a solid film (Figure 7c). Correspondingly, sulfur content increased from 14% at point B to 33% at point C and 46% at point D (**Figure** **8**a–c). At the middle charge state F, parts of the solid films were broken and sulfur particles were observed (Figure 7d). Until the end of the charge, no obvious solid films and sulfur particles were seen on the CNT surfaces (Figure 7e). Correspondingly, sulfur content decreased from 46% at point D to 25% at point F and 19% at point G (Figure 8d,e). The sulfur content in the SMCNT cathodes at various discharge and charge states was summarized in Figure 7f. In the discharge process, sulfur content decreased sharply at the high plateau, and then increased at the low plateau. In the charge process, sulfur content decreased continuously. As discussed previously, at the high discharge plateau, most of sulfur S_8_ should be transferred into soluble polysulfide species. While at the low discharge plateau, nonconductive Li_2_S_2_/Li_2_S materials were generated. During the subsequent charge process, these nonconductive Li_2_S_2_/Li_2_S materials dissolved into the liquid electrolytes and transformed into polysulfide species. According to the previous study on Li/S batteries, polysulfide species, when recharging after the very first discharge, do not transform back into elemental sulfur even at 100% depth of charge.^24^ Thus the 19% of sulfur at the end of charge should be ascribed to the remaining Li_2_S_2_/Li_2_S materials, although the contact films were destroyed. Combining these results with the EIS spectra from Figure 6b, we conclude that the formation and accumulation of nonconductive Li_2_S_2_/Li_2_S films were the main reason for rapid capacity fade in the liquid‐type Li–S batteries.

**Figure 9:**
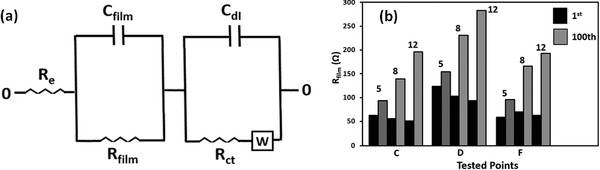
a) Equivalent circuit of the EIS spectra. b) *R*_film_ resistances of the solid Li_2_S_2_/Li_2_S films in the first cycle and 100th cycle with different quantity of electrolytes.

Based on the interpretation of the spectra of EIS, the equivalent circuit was constructed, as shown in **Figure** **9**a. In the equivalent circuits, *R*_e_ represents the impedance contributed by the resistance of the electrolyte, *R*_ct_ and *C*_dI_ are the charge transfer resistance at the conductive agent interface and its relative double‐layer capacitance, respectively. *R*_film_ and *C*_film_ are the resistance in the Li_2_S/Li_2_S_2_ film and its relative space charge capacitance, respectively. *W* is the Warburg impedance due to the diffusion of the polysulfides within the cathode.[[qv: 23a]] To further verify the above conclusion, we compared the resistances of the nonconductive Li_2_S_2_/Li_2_S films at the first cycle and the 100th cycle; the data from points C, D, and F were analyzed. From Figure 9b, the resistances in the first cycle were smaller than those in the 100th cycle for all of the tested points in the three different batteries. During the repeated cycles, the heat produced in the batteries and the side reactions would accelerate the evaporation and depletion of local electrolytes, and thus increased battery resistances. As can be seen, for the batteries with 12 μL mg^−1^ electrolytes, the rate of resistance increase at the two discharged states C and D was the fastest among the three different batteries. At the charged state F, the resistances were similar to the resistances at the middle discharged state C. This result indicated that at the middle charged state F, only some of the solid Li_2_S_2_/Li_2_S were transformed into soluble long‐chain polysulfide species. The other Li_2_S_2_/Li_2_S films became passivation layers and inhibited further lithiation/delithiation processes, which greatly increased battery resistance, and thus resulted in a rapid capacity fade.

Based on the EIS analysis and the morphology of the sulfur cathodes at different discharged and charged states, we believe that the formation and accumulation of solid Li_2_S_2_/Li_2_S films never stopped during the repeated cycles. In addition, sufficient electrolytes led to more deposition of Li_2_S_2_/Li_2_S films due to the enhanced sulfur reaction kinetics. However, the dissolution and transformation of Li_2_S_2_/Li_2_S to long‐chain polysulfide species were incomplete, thus with cycling, the Li_2_S_2_/Li_2_S films became thicker and thicker, which greatly reduced the migration of Li‐ion through the nonconductive films and hampered deeper discharge or charge in the batteries, and thus resulted in a rapid capacity fade.

## 4 Conclusion

In this study, a systematic capacity fade study was carried out for Li–S batteries employing different amounts of electrolytes. Three major causes of capacity fade including the loss of active sulfur materials into the liquid electrolyte (*Q*_1_), the precipitation of nonconductive Li_2_S_2_/Li_2_S films (*Q*_2_), and the incomplete conversions (*Q*_3_) were examined. In each case the precipitation of nonconductive Li_2_S_2_/Li_2_S films controlled the capacity fade of the battery. The thick Li_2_S_2_/Li_2_S films prohibited further lithiation processes and thus resulted in incomplete reactions. The loss of active sulfur materials into the liquid electrolytes directly influenced the discharge capacity from the second cycle. Sufficient electrolytes enhanced sulfur reaction kinetics, which was also favorable for the accumulation of thick Li_2_S_2_/Li_2_S films. EIS verified that the rapid capacity loss correlated with the increase of the internal resistance, which also resulted from the formation of thick Li_2_S_2_/Li_2_S films. SEM images further confirmed the deposition of such thick films during the repeated cycles on the surfaces of SMCNT cathodes. The formation kinetics of the solid nonconductive Li_2_S_2_/Li_2_S films and large sulfur agglomerates during cycling were also investigated: the semicircles in the middle frequency range were found to be caused by the solid Li_2_S_2_/Li_2_S films on CNT surfaces in the cathodes.

## 5 Experimental Section

*Fabrication of Binder‐Free SMCNT Cathodes*: The detailed information about the solvent exchange procedure to the pristine SMCNT materials in water was described in a previous work.^13^ The typical fabrication processes are described as follows: MCNTs were first refluxed in a mixture of concentrated nitric acid and sulfuric acid to remove amorphous carbon and to form oxygen‐containing groups on MCNT surfaces. Next, 55 mg of MCNTs together with a flat CNF paper (8 cm × 8 cm) current collector were treated with hydrogen peroxide (H_2_O_2_), and 122 mg of sulfur dissolved in carbon disulfide (CS_2_) solution was then added dropwise to form pristine SMCNTs on CNF paper. During the process, CS_2_ quickly reacted with H_2_O_2_ and formed colloidal sulfur. With this method, CNTs presented oxygen‐containing groups on their surfaces and sulfur was well dispersed on the CNT surfaces. Then, a freeze‐drying strategy was applied to the pristine SMCNT samples to achieve fine control of porous SMCNT framework structures and to form SMCNT cathodes.^25^ In brief, the pristine SMCNT/CNF samples were put onto a commercial CNF paper current collector, then the materials were frozen at low temperatures (typically −170 °C in liquid nitrogen) for 3 min. Frozen samples were then freeze‐dried using a Virtis automatic freeze‐dryer overnight to evaporate ice crystals. The sulfur loading in the SMCNT cathode was 1.9 mg cm^−2^, and sulfur content was 69%. The CNF current collector used in this experiment had an electrical conductivity of 420 S cm^−1^ and a thickness of ≈160 μm with a density of ≈0.2 g cm^−3^, corresponding to an areal CNF mass loading of 3.2 mg cm^−2^.

*SEM Tests*: The samples after cycling for SEM tests were prepared according to the following several steps. First, the disassembled cathodes were washed with DOL solution five times to remove the soluble polysulfides on the surfaces and bottoms of the cathode and anode. Next, the cathodes were left in the glove box for several hours to evaporate the remaining solution. Then, the cathode samples were anchored onto the SEM specimen mount holders, and placed into two separate vacuum jars for test. Finally, the samples were quickly transferred to the SEM chamber.

*Electrochemical Measurements*: CR2032‐type coin cells were used as the testing cells. Lithium foils were used as the anodes, Cellgard 2400 microporous membranes as separators, 1.0 mol L^−1^ bis(trifluoromethane sulfonyl) imide (LiTFSI) and 0.1 mol L^−1^ LiNO_3_ dissolved in DOL and 1,2‐dimethoxyethane (DME) (1:1, v/v) as electrolytes. The ring lithium foil with a thickness of 20 μm and diameter of 2.8 cm was used as the anode. LiNO_3_ was used to form a protective film on the surface of Li‐anode.^26^ The batteries were assembled in an argon‐filled glove box. The size of the cathode material was 1 cm × 1 cm. Electrochemistry measurements were performed galvanostatically between 1.8 and 3.0 V at various current densities. Capacity was calculated based on the weight of sulfur. CV experiments were conducted using a NOVA potentiostat at a scan rate of 0.1 mV s^−1^. EIS measurements were carried out using a NOVA electrochemical workstation in a frequency range between 100 kHz and 100 mHz at a potentiostatic signal amplitude of 5 mV. All experiments were conducted at room temperature.

## Supporting information

As a service to our authors and readers, this journal provides supporting information supplied by the authors. Such materials are peer reviewed and may be re‐organized for online delivery, but are not copy‐edited or typeset. Technical support issues arising from supporting information (other than missing files) should be addressed to the authors.

Supplementary
