# Supplementary material for: Capacity Fade Analysis of Sulfur Cathodes in Lithium–Sulfur Batteries
Source: Adv Sci (Weinh). 2016 Jul 21;3(12):1600101. doi: 10.1002/advs.201600101 (PMC5157169; doi:10.1002/advs.201600101)
Supplement: Supplementary file 1 — Supplementary [file ADVS-3-0-s001.pdf]

Copyright WILEY-VCH Verlag GmbH & Co. KGaA, 69469 Weinheim, Germany, 2016.

ADVANCED SCIENCE
Open Access

Supporting Information

for *Adv. Sci.,* DOI: 10.1002/advs.201600101

Capacity Fade Analysis of Sulfur Cathodes in Lithium–Sulfur Batteries

*Jianhua Yan, Xingbo Liu, and Bingyun Li**

Supporting Information

Capacity Fade Analysis of Sulfur Cathodes in Lithium Sulfur Batteries

Jianhua Yan[1, 2], Xingbo Liu[2], Bingyun Li[1*]

[1]*Biomaterials, Bioengineering & Nanotechnology Laboratory, West Virginia University, Morgantown, West Virginia 26506, United States. Email: bili@hsc.wvu.edu*

[2]*Department of Mechanical and Aerospace Engineering, West Virginia University, Morgantown, West Virginia 26506, United States.*

Characterization of Binder-Free SMCNT Cathodes

The porous SMCNT structure was synthesized by the facile two-step approach illustrated in **Figure S1a**. This strategy involved solvent exchange and freeze-drying techniques (see experimental section). Scanning electron microscopy (SEM) images of the composite, which had an irregular spherical morphology with CNTs protruding to the surface, are shown in **Figures S1b** and **S1c**. It was found that the infiltration of sulfur into MCNTs led to cathodes with minimized particle agglomeration. The average particle size was ~30 μm (**Figure S1d**). The cross-section image showed that the CNTs were dispersed throughout the particle interior (**Figure S1e**). XRD (**Figure S1f**) showed that sulfur in the SMCNT composite had both monoclinic phase and orthorhombic phase.[1] The monoclinic phase might attributed to the chemical bonds between sulfur and oxygen-containing groups on the CNT surfaces. The 3D CNF current collector (**Figure S1g**) might be of interest for enlarging the reaction area. In our previous study, we found this 3D current collector enabled good conductivity, and the flexible, porous architecture accommodated volume changes during cycling and retained the polysulfide species in the cathode region. In addition, the mechanical stability of the CNF current collector was sufficient to achieve long cycle life without binder.[2]

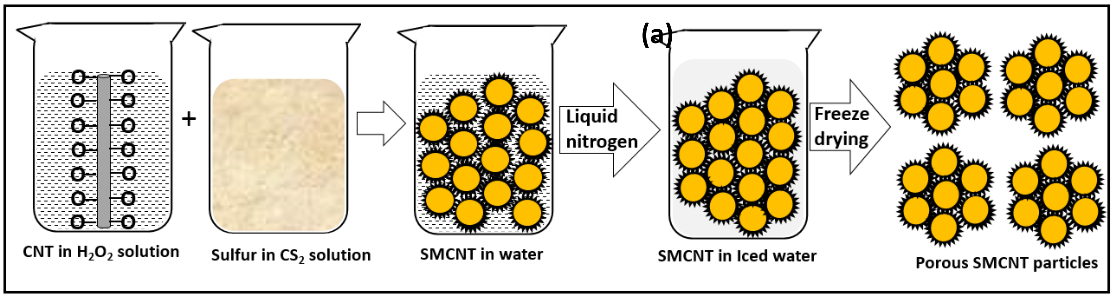

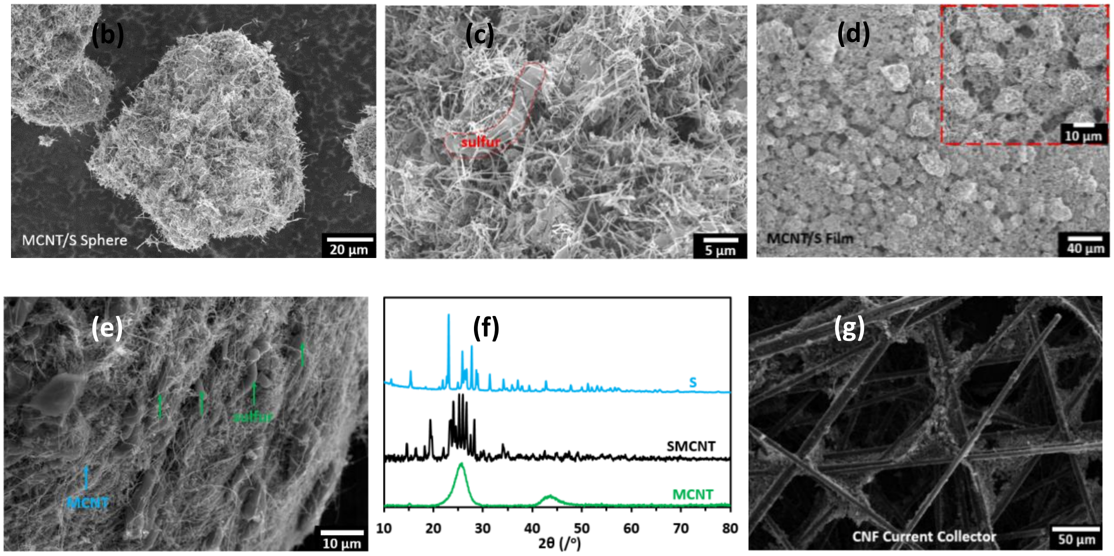

Figure S1. Procedures to form binder-free and porous SMCNT materials and related characterization. (**a**) Typical procedures to form SMCNT. (**b-d**) Surface and (**e**) cross-sectional SEM images of the porous SMCNT structures. (**f**) XRD analysis of SMCNT. (**g**) CNF current collector.

Reference

1. J. Yan, X. Liu, X. Wang, B. Li, *J. Mater. Chem. A* 2015, **3**, 10127.

2. a) J. Yan, X. Liu, H. Qi, W. Li, Y. Zhou, M. Yao, B. Li, *Chem. Mater.* 2015, **27**, 6394;

   b) J. Yan, X. Liu, B. Li, [C] *J. Electrochem. Soc.* 2015, **1**, 290.
